# ﻿Morphology, chemistry, and phylogeny reveal three new species of *Lecidea* (Lecideaceae) from China

**DOI:** 10.3897/mycokeys.121.161062

**Published:** 2025-09-02

**Authors:** Zhaojie Ren, Ruotong Li, Chunxiao Wang, Junxia Xue, Lisong Wang, Xinyu Wang, Lulu Zhang

**Affiliations:** 1 Shandong Museum, Jinan, 250014, China; 2 College of Geography and Environment, Shandong Normal University, Jinan, 250014, China; 3 State Key Laboratory of Phytochemistry and Natural Medicines, Kunming Institute of Botany, CAS, Kunming, 650201, China; 4 Yunnan Key Laboratory for Fungal Diversity and Green Development, Kunming Institute of Botany, CAS, Kunming, 650201, China

**Keywords:** East Asia, *Lecidea* s. str., lichen, Qinghai–Tibetan Plateau, taxonomy

## Abstract

Three new species, *Lecidea flavothallia*, *L. sublaboriosa*, and *L. tibetica*, are reported from China based on morphological, chemical, and molecular characters. *Lecidea flavothallia* is characterized by an orange thallus and the presence of schizopeltic acid. *Lecidea sublaboriosa* is characterized by a sparsely developed thallus, brown hypothecium, and the presence of 4-O-demethylplanaic acid in the apothecia. *Lecidea tibetica* is characterized by a well-developed thallus, I+ violet medulla, hyaline to pale straw-colored hypothecium, and the presence of 2′-O-methyperlatolic acid as the major secondary metabolite. All of the new species were collected from the Qinghai–Tibetan Plateau in Southwest China. Detailed descriptions, discussions, and figures are provided for each species, along with a key for all known Chinese *Lecidea* s. str. species.

## ﻿Introduction

*Lecidea* Ach. (Lecideaceae) was originally described by Acharius (1803). In the early stage, the classification of *Lecidea* was relatively broad, being characterized by crustose thalli, green algal photobionts, lecideine or biatorine exciples, and colorless ascospores (Spribille and Printzen 2007). With the advancement of research and the development of microscopy, the characteristics of ascospores, apothecia, and ascus apex structures were introduced into the classification, narrowing the scope of *Lecidea* (Massalongo 1852; Hafellner 1984). Hertel (1967, 1970, 1977a, 1977b, 1987, 1995, 2006) conducted a systematic study on *Lecidea*, further narrowing the taxonomic category of the genus. The species strictly in line with the definition of Hertel—as lichenologists call *Lecidea* s. str.—include only approximately 100 species. The main characteristics are thallus crustose to sub-squamulose; ascomata apothecia, lecideoid; asci 8-spored, *Lecidea*-type; ascospores simple, hyaline, without a perispore; and almost exclusively on rock (Hertel and Printzen 2004).

The Qinghai–Tibetan Plateau is approximately 2.5 million square kilometers (26°00′–39°47'N, 73°19′–104°47'E), including all of China’s Tibetan area and parts of Qinghai, Xinjiang, Gansu, Sichuan, and Yunnan provinces. It is the largest plateau in China and the highest in the world, known as the “Roof of the World.” Due to its special climatic, orographic, and geological conditions, the Qinghai–Tibetan Plateau is particularly rich in lichen species, but most of them are insufficiently understood.

During our study of the lichen flora of the Qinghai–Tibetan Plateau, three species of *Lecidea* were discovered, all of which are new to science. We present a brief diagnosis, an extended description, and a phylogenetic analysis based on ITS sequence data.

## ﻿Materials and methods

### ﻿Morphology and chemistry

The specimens were collected on the Qinghai–Tibetan Plateau, China, and are preserved in the Lichen Section of the Kunming Institute of Botany, Chinese Academy of Sciences (KUN). The specimens were examined morphologically using a COIC XTL7045B2 dissecting microscope and anatomically with an Olympus CX41 polarizing microscope and photographed under an Olympus SZX16 and BX61 microscope with a DP72 digital camera. The thallus, medulla, exciple, and epihymenium were tested with K (a 10% aqueous solution of potassium hydroxide), C (a saturated solution of aqueous sodium hypochlorite), or N (a 50% aqueous solution of nitric acid) for identification. Lugol’s iodine solution was employed to determine the amyloid reaction of the medulla and the type of asci. Crystals within the section were examined under polarized light. Lichen substances were identified using standardized thin-layer chromatography techniques (TLC) with systems B′ (hexane:methyl tert-butyl ether:formic acid = 140:72:18) and C (toluene:acetic acid = 200:30) (Orange et al. 2001; Elix 2014). In this study, *Lethariella cladonioides* (Nyl.) Krog, containing atranorin and norstictic acid, was used as the partition standard sample.

### ﻿Phylogenetic analyses

Total genomic DNA was extracted with a DNAsecure Plant Kit according to the manufacturer’s instructions and purified with a PCR quick-spin™ PCR Product Purification Kit. The ITS1–5.8S–ITS2 regions were amplified in a C1000™ automatic thermocycler using the primers ITS1F (Gardes and Bruns 1993) and ITS4 (White et al. 1990) in a 25 μL volume containing 12.5 μL of 2 × Taq PCR Master Mix (Aidlab) (containing Taq DNA polymerase: 0.1 unit/μL; MgCl_2_: 4 mM; and dNTPs: 0.4 mM), 8.5 μL of ddH_2_O, 2 μL of primer (1 μL of a 10 mM solution for each primer), and 2 μL of genomic DNA. PCR thermal cycling parameters were as follows: initial denaturation at 94 °C for 10 min, followed by 34 three-step cycles (95 °C for 45 s, 50 °C for 45 s, 72 °C for 1 min 30 s) and a final 10 min extension at 72 °C. PCR products were Sanger sequenced by Sangon Biotechnology Company.

All raw sequences were compared to those available in the GenBank database (http://www.ncbi.nlm.nih.gov/BLAST/) to ensure their reliability. The raw sequences were assembled and edited using SeqMan v. 7.0 (DNASTAR packages). Sequences extracted from the new materials were aligned with additional sequence data from GenBank using an online version of MAFFT v. 7.0.26 (Katoh et al. 2005). The MAFFT algorithm chose Auto (FFT-NS-1, FFT-NS-2, FFT-NS-i, or L-INS-i, depending on the data size). *Farnoldia jurana* (Schaer.) Hertel was chosen as the outgroup based on previous phylogenetic analyses.

Phylogenetic relationships were inferred using maximum likelihood (ML) and Bayesian inference (BI). ML was performed using the CIPRES Science Gateway (http://www.phylo.org/portal2/) (Miller et al. 2010). ML analysis was conducted using RAxML-HPC v. 8.2.12 (Stamatakis 2014), with default parameters as implemented on CIPRES, and support values were based on 1,000 non-parametric bootstrap pseudoreplicates. Bayesian analysis was conducted using MrBayes v. 3.2.7 (Ronquist et al. 2012). The best substitution models were estimated using ModelFinder (Kalyaanamoorthy et al. 2017). Based on the results, we used the TIM2 + F + G4 model for nrITS. Bayesian inference was performed with two parallel Metropolis-coupled Markov chain Monte Carlo runs (one “cold” chain and three heated chains), with trees sampled every 1,000 generations, and the run was automatically stopped when the average standard deviation of split frequencies fell below 0.01. The resulting tree was summarized after discarding the first 25% of samples. Generated phylogenetic trees were visualized using FigTree v. 1.4.2 (Rambaut 2012) and then edited in Adobe Illustrator (AI). Bootstrap support (BS) ≥ 70% and posterior probabilities (PP) ≥ 0.95 were considered significant supporting values and are presented on the tree.

## ﻿Results and discussion

**Figure 1.**
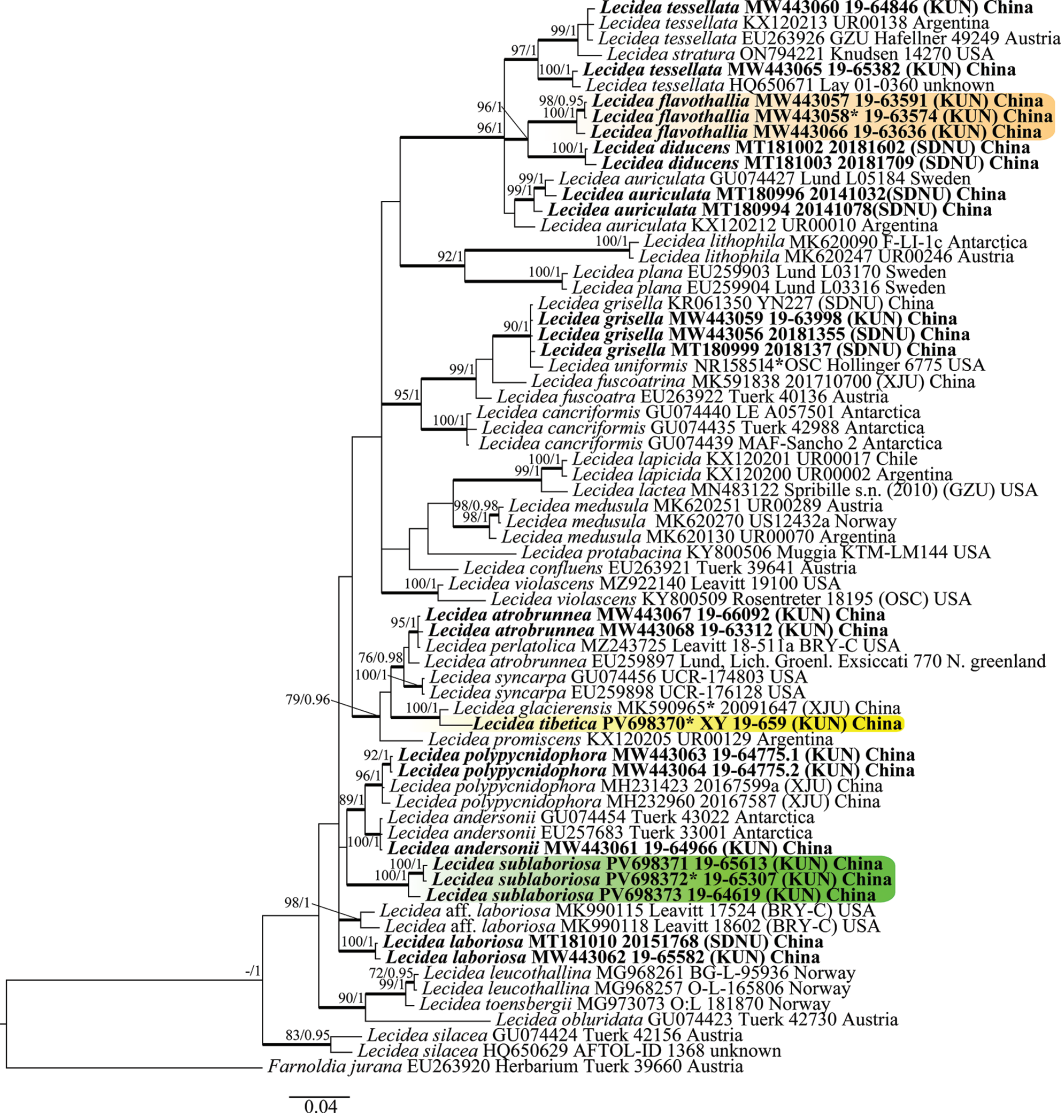
Phylogenetic tree constructed via Bayesian inference (BI) analysis of *Lecidea* species based on the concatenated nrITS dataset. Bootstrap support values ≥ 70 for ML and posterior probabilities ≥ 0.95 (second value) for Bayesian methods are indicated above the branches. Newly obtained sequences in this study are in bold; * represents type material. The new species are highlighted with colored backgrounds.

The sequence alignment comprised 69 nrITS sequences (46 downloaded from GenBank and 23 newly generated) of 33 taxa. The final dataset comprised a total of 469 sites. The phylogenetic trees obtained from ML and BI analyses exhibited a generally congruent topology; we therefore present only the BI tree, with BS ≥ 70% for the ML analysis and PP ≥ 0.95 for the Bayesian analysis (Fig. 1).

Although the results showed that the backbone of our tree is poorly supported, the three new species—*Lecidea flavothallia* C.X. Wang and Lu L. Zhang, *L. sublaboriosa* Z.J. Ren and Lu L. Zhang, and *L. tibetica* Z.J. Ren, Xin Y. Wang and Lu L. Zhang—formed monotypic lineages with strong support (100% ML, 1.00 PP) and were also supported by morphological and chemical characteristics. *Lecidea flavothallia* formed a well-supported clade (96/1.00) close to *L. diducens* Nyl.; however, *L. diducens* is characterized by a sparsely developed thallus and the presence of 2′-O-methylanziaic acid in the apothecial exciple (Hertel and Printzen 2004; Fryday et al. 2024). *Lecidea sublaboriosa*, *L. andersonii* R. Filson, and *L. polypycnidophora* U. Rupr. and Türk formed a distinct clade. Species of this clade produce either planaic/4-O-demethylplanaic acids or are acid deficient. Thallus development ranged from scant to largely endolithic; however, each species constitutes a monophyletic and strongly supported subclade. Additionally, these species can be distinguished based on morphological characteristics, which will be further discussed in the taxonomic descriptions. *Lecidea tibetica* appeared close to *L. glacierensis* A. Abbas and Mamut, but the two species can be separated by their external morphology and chemistry: *L. glacierensis* occurs on calcareous rock, has a non-amyloid medulla, smaller apothecia (0.4–1.5 mm), and contains confluentic acid as the major secondary metabolite (Mamut et al. 2022).

### ﻿Taxonomy

#### ﻿ Lecidea flavothallia

Taxon classificationFungiLecidealesLecideaceae

C.X. Wang & Lu L. Zhang sp. nov.

99FA6FDA-AC36-56D4-B22E-923AB75C15A3

838415

Fig. 2

##### Diagnosis.

Similar to *Lecidea truckeei* but with orange thallus and shorter conidia ([6–]7–9[–10.5] × 1 μm vs. [10–]11.5–15[–18] × 1.2 μm).

##### Type.

China, • Tibet Prov., Lazi Co., Xiqin vil., 29°04'24.14"N, 87°58'35.83"E, alt. 4492 m, on soil, 18 Jul. 2019, L.S. Wang et al. 19-63574 (KUN, holotype; GenBank MW443058).

##### Etymology.

The epithet “*flavothallia*” refers to the orange thallus.

##### Description.

Thallus: areolate, flat to bullate or lobate; prothallus: indistinguishable; areoles: dispersed, flat to convex, up to 2 mm in diam.; regular to irregular, angular to round in outline, with a whitish margin, esorediate; upper surface: shiny, (usually) yellowish orange to orange, occasionally with white or gray patches; cortex: 110–160(–220) μm thick; medulla: white, I–; algal layer: (60–)70–100(–150) μm thick; photobiont trebouxioid, cells (6–)7–10.5(–13) μm diam. Apothecia: abundant, subimmersed to sessile, lecideine, (0.6–)1.0–1.5(–2.0) mm diam.; disc: black, flat to convex, epruinose to faintly white pruinose; proper margin: black, prominent when young, occasionally becoming excluded in old apothecia. In section: exciple: black-brown outside, unpigmented inside, with small crystals (POL+) dissolving in N and insoluble in K; epihymenium: black-green to olive-green (N+ red-brown with a faint purple, K–), 10–15 μm thick; hymenium: hyaline or slight green, 40–60 μm tall; paraphyses: simple, occasionally scarcely branched and anastomosing; subhymenium: hyaline, 15–20 μm thick; hypothecium: brown, red-brown to dark brown; asci: clavate, *Lecidea*-type, 8-spored; ascospores: hyaline, simple, ellipsoid, (7–)8–9(–12) × 3.5–5 μm, length-width index: 1.8–2.2(–2.5) (n = 20). Conidiomata: immersed, graphidoid; conidia: bacilliform, (6–)7–9(–10.5) × 1 μm.

**Figure 2.**
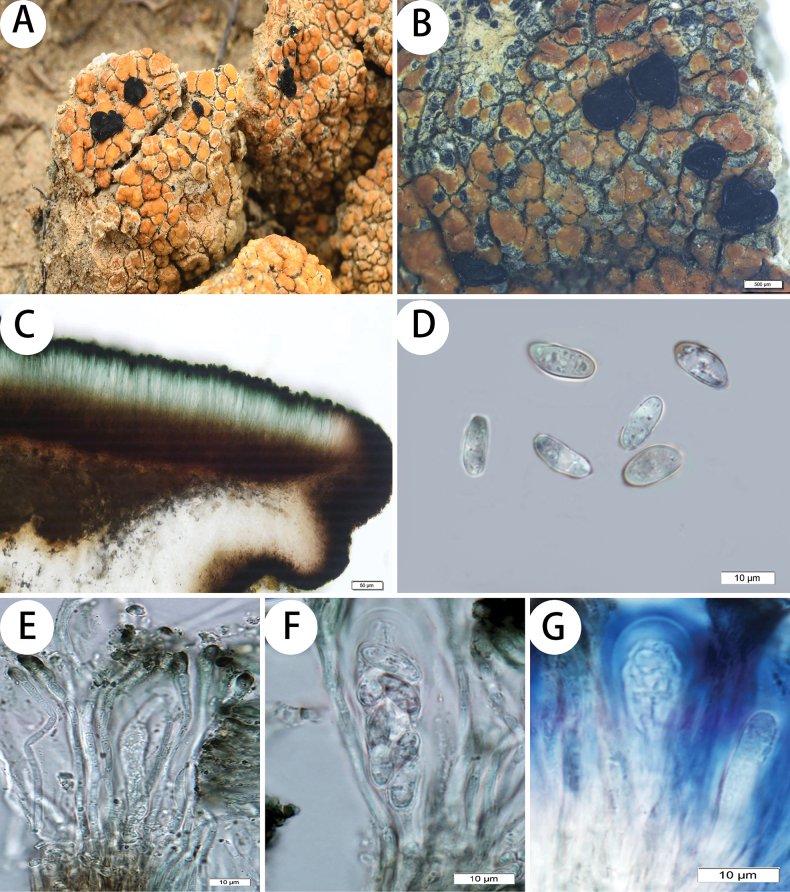
*Lecidea flavothallia*. A. Habitat and thallus (KUN 19-63574, holotype). (B–G KUN 19-63636): B. Apothecia; C. Apothecium section; D. Ascospores; E. Paraphyses; F. Ascus; G. Amyloid reaction of ascus. Scale bars: 500 µm (B); 50 µm (C); 10 µm (D, E, F, G).

##### Chemistry.

Cortex and medulla K+ yellow, C–, KC–; schizopeltic acid detected by TLC.

##### Additional specimens examined.

China • Tibet Prov., Lazi Co., Xiqin vil., 29°04'18.92"N, 87°58'37.49"E, alt. 4536 m, on soil, 18 Jul. 2019, L.S. Wang et al. 19-63591 (KUN); 19-65264 (KUN). • Angren Co., Kerangla vil., 29°19'01.67"N, 87°01'58.50"E, alt. 4530 m, on siliceous rock, 19 Jul. 2019, L.S. Wang et al. 19-63636 (KUN). • Dingri Co., Zhaguo vil., 28°35'09.72"N, 87°03'44.35"E, alt. 4316 m, on siliceous rock, 16 Jun. 2022, L.S. Wang et al. 22-71230 (KUN). • Jilong Co., Zheba vil., 29°17'25.50"N, 85°14'52.99"E, alt. 4555 m, on siliceous rock, 17 Jun. 2022, L.S. Wang et al. 22-71328 (KUN); • 29°17'24.62"N, 85°14'53.17"E, alt. 4571 m, on siliceous rock, 17 Jun. 2022, L.S. Wang et al. 22-71332 (KUN). • Yunnan Prov., Deqin Co., Baima Snow Mountain, 28°20'513"N, 99° 03'984"E, alt. 4320 m, on soil, 28 Aug. 2006, L.S. Wang, Oh Soon-Ok & Niu Dong-ling. 06-26744 (KUN).

##### Distribution.

Currently, this species is only found on the Qinghai-Tibetan Plateau of China, growing on exposed rocks and occasionally on alpine meadow permafrost soil, between altitudes of 4,000 and 5,000 m.

##### Discussion.

The species has the general appearance of a member of the *Lecidea atrobrunnea* group, but the I– medulla and short conidia indicate otherwise. Molecular data indicate that it belongs to the *L. auriculata* group, with which it shares the dark hypothecium, low hymenium, narrow ascospores, and graphidioid conidiomata, but that it differs from other members of this group by the atrobrunnea-type thallus containing schizopeltic acid. Other *Lecidea* species with schizopeltic acid include *L. cinerata* Zahlbr., *L. hassei* Zahlbr., *L. mannii* Tuck., and *L. truckeei* Herre, but *L. cinerata* has larger ascospores (13–16 × 4.2–6.3 µm vs. [7–]8–9[–12] × 3.5–5 μm) and a C+ red exciple (lecanoric acid vs. schizopeltic acid); *L. hassei* has an endolithic thallus and a brownish opaque exciple; *L. mannii* has a black prothallus, larger ascospores (10–15 × 5–6.7 µm vs. [7–]8–9[–12] × 3.5–5 μm), and gyrophoric acid (C+ red); and *L. truckeei* has a dark reddish brown thallus and an I+ intensely violet medulla (Herre 1911; Hertel and Printzen 2004; Lendemer and Knudsen 2007; Fryday and Hertel 2014). Additionally, *Lecidea flavothallia* and *L. poeltii* Hertel share similar characteristics, both possessing a shiny, well-developed thallus, a green epihymenium, a low hymenium, and a dark hypothecium, with distributions in the Himalayan region. However, *L. poeltii* differs in having a brown thallus, I+ medulla, and larger mature spores (11–16 × 4.5–7 µm vs. [7–]8–9[–12] × 3.5–5 μm) (Hertel 1975, 1977a).

#### ﻿ Lecidea sublaboriosa

Taxon classificationFungiLecidealesLecideaceae

Z.J. Ren & Lu L. Zhang sp. nov.

E4C3359D-B496-52FF-BF20-1C07B0A58CA1

859348

Fig. 3

##### Diagnosis.

Similar to *Lecidea laboriosa* but with a brown to dark brown hypothecium and shorter ascospores ([6–]7–8.5[–10] × 2.5–3.5[–4] μm vs. [6–]8–12[–16] × [2–]2.5–4[–5] µm).

##### Type.

China • Tibet Prov., Angren Co., Sangsang vil., 29°18'59.01"N, 87°02'02.97"E, alt. 4498 m, on rock, 19 Jul. 2019, L.S. Wang et al. 19-65307 (KUN, holotype; GenBank PV698372).

##### Etymology.

The specific epithet refers to the new species’ similarity to *Lecidea laboriosa*.

##### Description.

Thallus: endolithic to scarcely epilithic; prothallus: absent; surface: if present, whitish to cream-colored, rough, esorediate; medulla: white, I– or partially weakly I+ violet; algal layer: not obvious; photobiont trebouxioid, cells (9–)10–13(–15) μm diam. Apothecia: abundant, sessile, lecideine, 0.65–1.5 mm diam.; disc: black, flat to convex, epruinose to faintly white pruinose; proper margin: black, regular or corrugated, occasionally becoming excluded in old apothecia. In section: exciple: black-brown outside, hyaline to light brown inside, with small crystals (POL+) dissolving in N and insoluble in K; epihymenium: black-green (N+ violaceous, K–), 7.5–12.5 μm thick; hymenium: hyaline or slight blue-green, 30–40 μm tall; paraphyses: simple, unbranched, slightly enlarged at the apex, apical cells 2.5–3(–4) μm; subhymenium: hyaline to light brown, 12–20 μm thick; hypothecium: brown to dark brown; asci: clavate, *Lecidea*-type, 8-spored; ascospores: hyaline, simple, ellipsoid, (6–)7–8.5(–10) × 2.5–3.5(–4) μm, length-width index: 1.8–2.5(–3.2) (n = 20). Conidiomata: not seen.

##### Chemistry.

Cortex and medulla K–, C–, KC–; 4-*O*-demethylplanaic acid detected by TLC.

##### Additional specimens examined.

China • Tibet Prov., Zhongba Co., Larang Vil., 29°47'20.00"N, 83°54'12.10"E, alt. 4652 m, on rock, 25 Jul. 2019, L.S. Wang et al. 19-64947 (KUN); 19-65613 (KUN). • Dazi Dist., Bangdui Vil., 29°45'44.00"N, 91°25'29.28"E, alt. 3698 m, on siliceous rock, 16 Jul. 2019, L.S. Wang et al. 19-64619 (KUN).

##### Distribution.

This species is found in the plateau sub-frigid, semi-arid climate zone of China, growing on exposed siliceous rocks.

##### Discussion.

We have conducted multiple phylogenetic tree reconstructions, in which *Lecidea sublaboriosa* is placed as a sister species to *L. laboriosa* Müll. Arg. is either weakly supported or unsupported. Despite this phylogenetic uncertainty, *L. sublaboriosa* exhibits a high degree of morphological and chemical similarity with *L. laboriosa*, including an endolithic to scarcely epilithic thallus, a green epihymenium, a low hymenium, and the presence of 4-*O*-demethylplanaic acid. Additionally, it is morphologically similar to *L. andersonii*, *L. auriculata* Th. Fr., *L. diducens*, and some specimens of *L. polypycnidophora*, in that they all either lack an epilithic thallus or, if one is present, it is poorly developed. All of the above species have been detected in the Qinghai-Tibetan Plateau; however, they differ in that *Lecidea andersonii* has larger ascospores ([8–]9–12 × 3.5–4.4 μm vs. [6–]7–8.5[–10] × 2.5–3.5[–4] μm) and an I+ violet medulla; *L. auriculata* has an I+ violet medulla and contains confluentic acid; *L. diducens* has a C+ red exciple and contains 2’-*O*-methylanziaic acid; *L. laboriosa* has larger ascospores ([6–]8–12[–16] × [2–]2.5–4[–5] µm vs. [6–]7–8.5[–10] × 2.5–3.5[–4] μm) and hyaline to light brown hypothecium; *L. polypycnidophora* has broader ascospores (6–8 × 3–4.5 μm vs. [6–]7–8.5[–10] × 2.5–3.5[–4] μm) and abundant pycnidia (Inoue 1991; Hertel and Printzen 2004; Ruprecht et al. 2010; Fryday et al. 2024).

**Figure 3.**
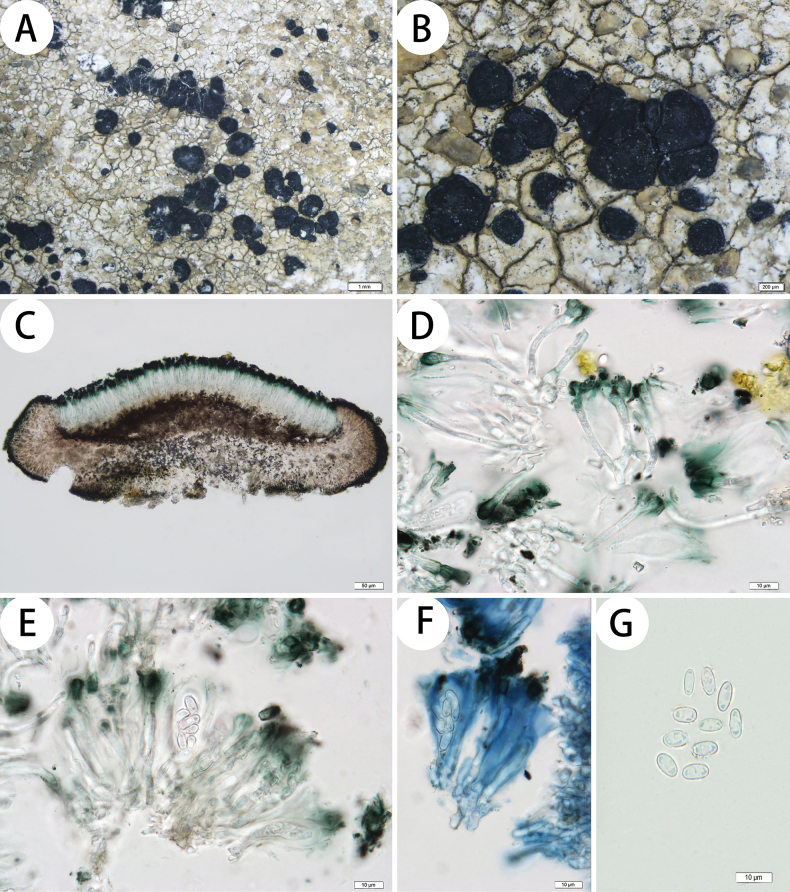
*Lecidea sublaboriosa* (KUN 19-65307, holotype). A. Thallus; B. Apothecia; C. Apothecium section; D. Paraphyses; E. Ascus; F. Amyloid reaction of ascus; G. Ascospores. Scale bars: 1 mm (A); 200 µm (B); 50 µm (C); 10 µm (D, E, F, G).

#### ﻿ Lecidea tibetica

Taxon classificationFungiLecidealesLecideaceae

Z.J. Ren, Xin Y. Wang & Lu L. Zhang sp. nov.

7BF91E62-1DC1-51D2-8D90-C018B6E4FCF1

859349

Fig. 4

##### Diagnosis.

*Lecidea tibetica* is characterized by a well-developed thallus, I+ violet medulla, hyaline to pale straw-colored hypothecium, and the presence of 2’-*O*-methyperlatolic acid as the major secondary metabolite.

##### Type.

China • Tibet Prov., Chayu Co., Zhuwagen Town Mont. Pass, 28°43'17.75"N, 97°42'21.01"E, alt. 4685 m, on siliceous rock, 13 Jul. 2019, X.Y. Wang et al. XY 19-659 (KUN, holotype; GenBank PV698370).

##### Etymology.

The epithet “*tibetica*” refers to the type location, Tibet Province, China.

##### Description.

Thallus: crustose, areolate to rimose-areolate, flat to bullate, thick (up to 1.1 mm); prothallus: black, obvious at the margin of the thalli; areoles: dispersed, irregular, flat to convex, sometimes expand into a tuberous structure; surface: gray to bluish gray or whitish gray (the shady side), esorediate; cortex: 12–20 μm thick; medulla: white, I+ violet; algal layer: 50–100 μm thick; photobiont trebouxioid, cells (5.5–)7.5–13 μm diam. Apothecia: black, immersed to subimmersed, lecideine, up to 4.5 mm in diam.; disc: black, flat to slightly convex, epruinose, often irregularly cracked in old apothecia; proper margin: dull, often dark grey, persistent, sometimes undulate. In section: exciple: dark brown outside, unpigmented inside, with large crystals insoluble in K and N; epihymenium: olive-brown (N+ orange-brown, K–), 15–25 μm thick; hymenium: hyaline, 70–85 μm tall; paraphyses: simple, occasionally scarcely branched and anastomosing; subhymenium: hyaline, poorly distinguishable from the hymenium; hypothecium: hyaline to pale straw colored, c. 200 μm; asci: clavate, *Lecidea*-type or *Porpidia*-type (occasionally observed in immature asci), 8-spored; ascospores: hyaline, simple, ellipsoid, 11.5–13 × 6–8 μm, length-width index: 1.6–2 (n = 20). Conidiomata: immersed, graphidoid; conidia: bacilliform, (14–)15–18(–19) × 1 μm.

##### Chemistry.

Cortex and medulla K+ yellow, C–, KC–; thallus UV–; 2’-*O*-methyperlatolic acid and confluentic acid (trace) were detected by TLC.

##### Additional specimens examined.

China • Tibet Prov., Chayu Co., Yixiula Puerto, 28°44'16.62"N, 97°42'7.98"E, alt. 4492 m, on siliceous rock, 25 Sep. 2014, L.S. Wang et al. 14-46952 (KUN).

##### Distribution.

This species is associated with *Rhizocarpon* sp. and has been documented so far only in the Tibet Province of China, where it grows on non-calcareous rocks in high-altitude areas.

##### Discussion.

*Lecidea tibetica* is morphologically similar to *L. confluens* (Weber) Ach., *L. tessellata* Flörke, and *Porpidia speirea* (Ach.) Kremp., as they all have a well-developed and grayish thallus, I+ violet medulla, and immersed to sub-immersed apothecia. However, among the latter three species, all exhibit smaller apothecia (generally less than 2 mm), conidia measuring 8–13 × 1–1.3 μm (vs. [14–]15–18[–19] × 1 μm), and contain confluentic acid as the primary secondary metabolite. Specifically, *Lecidea confluens* has a brown to dark brown hypothecium, while *L. tessellata* lacks conspicuous apothecial margins. *Porpidia speirea* can be distinguished by the presence of prominent dark parathecial margins, halonate ascospores, and a dark brown hypothecium. Like *Lecidea tibetica*, *L. stratura* K. Knudsen & Lendemer also has a white to grayish thallus, I+ medulla, and contains 2’-*O*-methyperlatolic acid. However, it has a blue to blue-green epihymenium, a shorter hypothecium (70–100 μm vs. 200 μm), smaller ascospores (8–9.5 × 3.0–5.5 μm vs. 11.5–13 × 6–8 μm), and a distinctly thin thallus (usually 0.2–0.5 mm thick) rather than a thick thallus (up to 1.1 mm thick) like *L. tibetica* (Knudsen et al. 2017).

**Figure 4.**
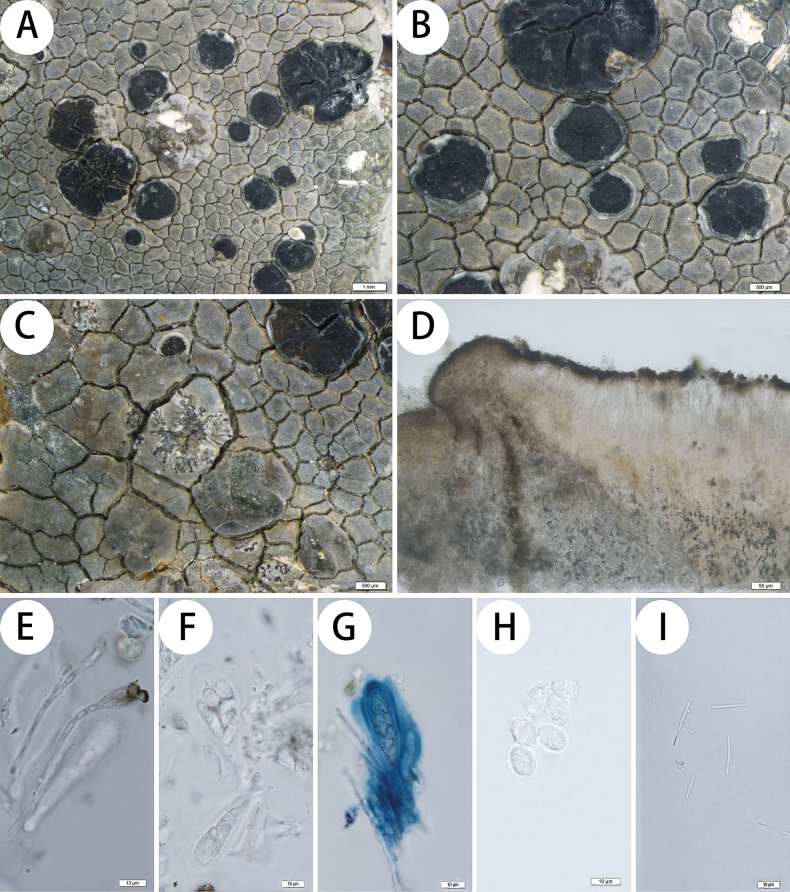
*Lecidea tibetica* (KUN XY 19-659, holotype). A. Thallus; B. Apothecia; C. Conidiomata; D. Apothecium section; E. Paraphyses; F. Ascus; G. Amyloid reaction of ascus; H. Ascospores; I. Conidia. Scale bars: 1 mm (A); 500 µm (B, C); 50 µm (D); 10 µm (E, F, G, H, I).

## ﻿Conclusion

The Qinghai–Tibetan Plateau features an average elevation exceeding 4,000 meters. It encompasses a wide range of ecosystems, extending from forests and grasslands to deserts, thereby supporting a vast and heterogeneous territory with diverse habitats. This distinctive geographical environment has fostered the development of unique and highly varied lichen species. Species of *Lecidea* s. str. are predominantly distributed in alpine regions and are commonly found within the altitude range of 3,000–5,500 meters on the Qinghai–Tibetan Plateau. Growing on exposed rock surfaces, they often coexist with various lichen taxa, forming a variegated “carpet.”

Currently, 23 species of the genus *Lecidea* s. str. have been reported from China, with 16 of these species detected on the Qinghai–Tibetan Plateau (Magnusson 1940, 1944; Hertel 1977a; Zhang et al. 2010, 2012; Hu et al. 2014; Zhao et al. 2017; Jiamahat and Mamut 2019; Wei 2020; Mamut et al. 2022). However, due to the relatively short history of lichen research in China, a considerable number of intriguing species have yet to be documented. Supported by relevant national funding projects and the Second Tibetan Plateau Scientific Expedition and Research Program, in-depth investigations and studies on the lichen biodiversity of the Qinghai–Tibetan Plateau are gradually revealing the complex species composition of the genus *Lecidea* in this area.

### ﻿Key to the species of *Lecidea* s. str. in China

**Table:** 

1	Thallus thin, absent to patchy	**2**
–	Thallus well developed, conspicuous	**11**
2	No secondary metabolites detected by TLC	**3**
–	Secondary metabolites detected by TLC	**5**
3	Hypothecium hyaline to light brown	***Lecidea paratropoides***
–	Hypothecium brown to dark brown	**4**
4	Most apothecia > 0.5 mm diam., the largest ones up to 4 mm; medulla I+ violet	***Lecidea promiscens***
–	Most apothecia < 0.5 mm diam., the largest ones up to 1 mm; medulla I–	***Lecidea promixta***
5	Stictic acid is a major lichen substance (norstictic acid lacking or minor stictic acid chemosyndrome)	***Lecidea lapicida***
–	Stictic acid or norstictic acid not detectable	**6**
6	2’-*O*-methylanziaic present in apothecia; excipulum C+ red	***Lecidea diducens***
–	2’-*O*-methylanziaic not detectable in apothecia; excipulum C–	**7**
7	Confluentic acid is the main lichen substance	**8**
–	4-*O*-demethylplanaic is the main lichen substance	**9**
8	Ascospores small (6–11 × 2.5–3.5 µm); excipulum rather broad, often becoming flexuous	***Lecidea auriculata***
–	Ascospores larger (6.5–12 × 3.3–4.5 µm); excipulum narrower, usually not markedly flexuous	***Lecidea promiscens***
9	Medulla I+ violet, excipulum rather broad in young apothecia	***Lecidea andersonii***
–	Medulla I– or I+ pale violet, excipulum normal-sized in young apothecia	**10**
10	Ascospores small (7–8.5 × 2.5–3.5 μm), hypothecium brown to dark brown	***Lecidea sublaboriosa***
–	Ascospores larger (8–12 × 2.5–4 µm), hypothecium hyaline to light brown	***Lecidea laboriosa***
11	Schizopeltic acid detected, thallus yellowish orange to orange	***Lecidea flavothallia***
–	Schizopeltic acid not detected	**12**
12	Stictic acid or hypostictic acid detected	***Lecidea protabacina***
–	Stictic acid and hypostictic acid not detectable	**13**
13	Norstictic acid detected	**14**
–	Norstictic acid not detectable	**15**
14	Thallus thin or moderately thick, areolate plane, white to bluish-gray	***Lecidea lactea***
–	Thallus crustose, bullate to subsquamulose, pale yellowish brown to more rarely dark brown (atrobunnea-type)	***Lecidea syncarpa***
15	Gyrophoric acid detected by TLC	**16**
–	Gyrophoric acid not detectable by TLC	**19**
16	Ascospores usually longer than 10 μm	**17**
–	Ascospores usually shorter than 10 μm	**18**
17	Thallus at the margins starting as confluent areoles, surface grey-brown to red-brown	***Lecidea fuscoatra***
–	Thallus continuous at the margins, rimose to areolate towards the center, surface grey	***Lecidea grisella***
18	Thallus thin (ca 0.1 mm thick), shiny; apothecia margin persistent	***Lecidea siderolithica***
–	Thallus thick (up to 5 mm thick), dull; old apothecia without conspicuous margin and often irregularly cracked	***Lecidea sinensis***
19	4-*O*-demethylplanaic acid detected by TLC	**20**
–	4-*O*-demethylplanaic acid not detectable by TLC	**21**
20	Apothecia black or pruinose, discs turn brown on wetting, epithecium brown, hypothecium colorless	***Lecidea lithophila***
–	Apothecia black, not pruinose; epithecium dark green, hypothecium brown; pycnidia abundant	***Lecidea polypycnidophora***
21	Perlatolic acid detected by TLC; thallus pale to medium brown to (more rarely) dark brown in the center	***Lecidea perlatolica***
–	Perlatolic acid not detectable by TLC	**22**
22	2’-*O*-methyperlatolic acid is the main lichen substance; apothecia large, up to 4.5 mm in diam.; hypothecium hyaline to pale straw colored; medulla I+ violet	***Lecidea tibetica***
–	Confluentic acid is the main lichen substance	**23**
23	Thallus brown, with an epinecral layer (atrobrunnea-type)	**24**
–	Thallus white to grey, lacking an epinecral layer	**25**
24	Ascospores small (7.5–10 × 2.5–5 µm), hypothecium hyaline to brown	***Lecidea atrobrunnea***
–	Ascospores larger (10–17 × 4.5–7.5 µm), hypothecium dark brown to almost black	***Lecidea fuscoatrina***
25	Apothecia margin very thin or disappearing, hypothecium hyaline to very pale green or pale yellowish brown	***Lecidea tessellata***
–	Apothecia margin usually persistent, hypothecium brownish	**26**
26	Ascospores 6.5–7.5 μm wide, medulla I+ violet	***Lecidea confluens***
–	Ascospores 7–9 µm wide, medulla I–	***Lecidea glacierensis***

## Supplementary Material

XML Treatment for Lecidea flavothallia

XML Treatment for Lecidea sublaboriosa

XML Treatment for Lecidea tibetica

## ﻿Additional information

### Use of AI

No use of AI was reported.

### Author ORCIDs

Zhaojie Ren https://orcid.org/0000-0003-3206-1484

Ruotong Li https://orcid.org/0009-0003-9428-3055

Chunxiao Wang https://orcid.org/0009-0009-2094-4854

Junxia Xue https://orcid.org/0000-0002-6387-0148

Lisong Wang https://orcid.org/0000-0003-3721-5956

Xinyu Wang https://orcid.org/0000-0003-2166-6111

Lulu Zhang https://orcid.org/0000-0001-8011-4451

### Data availability

All of the data that support the findings of this study are available in the main text.
